# The hidden secrets of a neutral pH—blood gas analysis of postoperative patients according to the Stewart approach

**DOI:** 10.1186/s13741-021-00186-4

**Published:** 2021-06-08

**Authors:** Joost W. Janssen, Joris M. K. van Fessem, Tijmen Ris, Robert Jan Stolker, Markus Klimek

**Affiliations:** Department of Anesthesiology, Erasmus University Medical Center, Dr. Molewaterplein 40, 3015 GD Rotterdam, The Netherlands

**Keywords:** Acid-base balance, Stewart analysis, Postoperative period, Hyperchloremia, Hypoalbuminemia

## Abstract

**Background:**

The superiority of either the traditional or Stewart based approach to acid-base balance has focused primarily on analyzing metabolic acidemia, with little attention given to patients with neutral pH. In this study, we evaluate metabolic disturbances in patients in the immediate postoperative period focusing on patients with neutral pH, while comparing the Stewart and traditional approach.

**Methods:**

We conducted a single center retrospective observational cohort study. Over a 17-month period, data on arterial blood gas analysis, electrolytes, and albumin on the morning after surgery were retrieved from patients admitted to the postsurgical high dependency unit (HDU). Albumin-corrected anion gap (AG), apparent (SIDa) and effective strong ion difference (SIDe), and strong ion gap (SIG) were calculated.

**Results:**

Out of 1207 HDU admissions, 400 cases had a complete set of laboratory-data including albumin of which 281 presented with neutral pH (7.35 ≤ pH ≤ 7.45), 64 with acidemia (pH < 7.35) and 55 with alkalemia (pH > 7.45). In pH neutral patients, the following acidifying disturbances were found: SIDa was lowered in 101 (36%), and SIG was raised in 60 (21%). Base excess (BE) was decreased in 16 (6%) and corrected AG raised in 107 (38%). The alkalizing effect of hypoalbuminemia was present in 137 (49%). Out of 134 cases with normal BE and corrected AG, SIDa was lowered in 58 (43%). Out of 136 cases with normal SIDa and SIG, none had lowered BE and 28 increased AG (21%). Length of stay was significantly longer in patients with hypoalbuminemia, lowered SIDa, and increased corrected AG, but not decreased BE (hypoalbuminemia: 16 days vs. 10 days, *P* < 0.001; low SIDa: 15 days vs. 12 days, *P* = 0.015; increased AG: 16 days vs. 11 days, *P* < 0.001; low BE: 14 days vs. 13 days, *P* = 0.736).

**Conclusions:**

Metabolic disturbances, characterized mainly by the presence of lowered SIDa, increased AG, and hypoalbuminemia, are frequent in our population with apparent neutral acid-base balance based on pH and base excess. These changes on the morning after surgery are associated with increased length of stay.

## Background

Acid-base disturbances are common in patients undergoing major surgery and therefore arterial blood gas (ABG) analysis is frequently performed in the perioperative period. When evaluating a patient’s acid-base status, clinicians often rely on parameters based on the traditional Henderson-Hasselbalch and Siggaard-Andersen approach as provided by the blood gas analyzer: pH, pCO_2_, HCO_3_^−^, and base excess (BE), with a patient being classified as acid-base neutral if all parameters fall within reference range. An alternative approach, which has gained popularity especially in the critical care community, is the Stewart approach. According to Stewart, acid-base balance is influenced by three independent variables: pCO_2_, strong ion difference (SID) and the total of non-volatile weak acids (mainly albumin and phosphate). For detailed information on the Stewart approach, we refer to other resources (Stewart 1983; Fencl and Leith 1993).

Previous studies, predominantly performed in an intensive care unit (ICU) population, have shown inconsistent results in the superiority of either the traditional or Stewart approach in diagnosing metabolic acidemia (Kimura et al. 2018). However, much less attention has been given to metabolic disturbances in those patients with a neutral pH. The goal of this study is first to identify and classify metabolic disturbances comparing the traditional and Stewart approach in the early postoperative period in patients presenting with neutral pH and comparing this to disturbances found in patients with acidemia and alkalemia. Secondly, we look whether the disturbances found using the Stewart approach in these patients are associated with relevant clinical outcomes.

## Methods

This retrospective observational cohort study was conducted at the Erasmus University Medical Center in Rotterdam, The Netherlands. All patients admitted to the postsurgical high-dependency unit (HDU) of the Erasmus Medical Center in the period of June 1, 2018, until October 31, 2019, were eligible for inclusion. The HDU in our hospital has intensive care facilities and provides care in the first 24 postoperative hours for patients who underwent major surgery and/or high risk patients. During HDU admission, ABG and electrolyte analysis (pH, pCO_2_, lactate, sodium, chloride and potassium) is routinely performed on the morning after surgery using a point-of-care blood gas analyzer (Radiometer ABL90 Flex Plus, Copenhagen, Denmark). Additional laboratory measurements including albumin, creatinine, phosphate, magnesium, and calcium are performed on a separate blood sample when deemed appropriate by the attending physician. Cases were excluded if ABG and/or albumin were not measured on the morning after surgery. If calcium, magnesium, and/or phosphate measurements were unavailable, normal values were imputed based on average local laboratory reference values ([Ca^*2+*^] = 2.4 mmol/L, [Mg^*2+*^] = 0.9 mmol/L, [Phosphate] = 1.1 mmol/L). Additional patient characteristics, i.e., age, sex, weight, surgical blood loss, intra- and postoperative fluid therapy up to the morning after surgery, indication for HDU admission, American Society of Anesthesiologists (ASA) physical status classification, length of stay from day of surgery until discharge, and mortality were obtained. The following infusion fluids are used in our hospital: Sterofundin® (B. Braun, Melsungen, Germany) as balanced and 0.9% NaCl as unbalanced crystalloid, Volulyte® as balanced and Voluven® as unbalanced colloid (both Fresenius Kabi, Huis ter Heide, Netherlands).

### Calculations

For the traditional approach, the serum bicarbonate concentration was calculated using the Henderson-Hasselbalch equation and BE using the Van Slyke equation (Siggaard-Andersen et al. 1988). To augment the traditional approach, the anion gap (AG) was calculated using AG = [Na^+^] + [K^+^] − [Cl^−^] − [HCO_3_^−^] and corrected for albumin to improve its accuracy using AG + 0.25 × (40 − [albumin]) (Figge et al. 1998). For the Stewart approach, the following calculations were used based on the work of Stewart (Stewart 1983) and Figge (Figge et al. 1992):

- Apparent strong ion difference (SIDa) = [Na^+^] + [K^+^] + [Ca^2+^] + [Mg^2+^] − [Cl^−^] − [lactate]
- Effective strong ion difference (SIDe) = [HCO_3_^−^] + ([albumin] × 0.123 × (pH − 0.631)) + ([phosphate] × (0.309 × pH − 0.469))
- Strong ion gap (SIG) = SIDa − SIDe

All values in mmol/L with exception of albumin in g/L.

### Definitions

In our hospital, the following reference ranges are used: pH 7.35–7.45, pCO_2_ 4.7–6.4 kPa, HCO_3_^−^ 21–27 mmol/L, BE − 3–3 mmol/L, chloride 97–107 mmol/L, and sodium 135–145 mmol/L. The reference range for SIDa is established as 38–42 mmol/L (Gunnerson and Kellum 2003; Gunnerson et al. 2010; Nagaoka et al. 2010; Park et al. 2008). In order to provide a range equal to the values used for HCO_3_^−^ and BE, we considered SIDa values of 37–43 mmol/L as normal. Hypoalbuminemia was defined as albumin < 30 g/L, hyperlactatemia as lactate > 2 mmol/L, hyperphosphatemia as phosphate > 2 mmol/L, increased AG as AG > 12 mmol/L, and increased SIG as SIG > 2 mmol/L. Decreased kidney function was defined as serum creatinine > 150 μmol/L (Jones et al. 1998). Patients were grouped according to their pH: acidemia (pH < 7.35), neutral (7.35 ≤ pH ≤ 7.45), and alkalemia (pH > 7.45). Data are represented as mean ± standard deviation when appropriate. Primary outcome was the incidence of acid-base disturbances according to the traditional and Stewart approach across three pH categories. Secondary outcomes were length of stay (LOS), acute kidney injury (AKI), defined as a postoperative twofold increase in serum creatinine, pulmonary embolism, myocardial infarction, unplanned ICU admission, and mortality. For statistical analysis, we used the unpaired Students *t* test. The diagnostic performance of BE and SIDa for prediction of dichotomous secondary outcomes was evaluated by calculating the area under the curve (AUC) of the receiver operating characteristic (ROC) curves. A *P* value < 0.05 was considered significant.

## Results

**Fig. 1:**
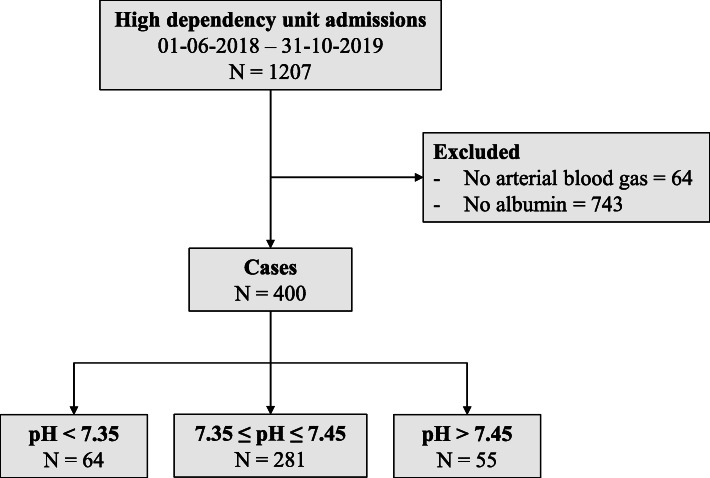
Flowchart of patients included in the study

A total of 400 patients were included in the study of which 64 presented with acidemia, 55 with alkalemia, and 281 with neutral pH the morning after surgery (Fig. 1). Patient characteristics, cumulative intra- and postoperative intravenous fluid therapy, outcome measures, and acid-base data are summarized in Table 1. Serum creatinine was measured in 395 patients of which 43 showed evidence of decreased kidney function. Phosphate was measured in 270 cases, calcium in 221 cases, and magnesium in 365 cases. Mean time after surgery, at which time point laboratory measurements took place, was 18 ± 10 h. Table 2 presents an overview of respiratory and metabolic changes detected by using the Stewart and traditional approach.

**Table 1:** Patient characteristics and acid-base parameters

	pH < 7.35	7.35 ≤ pH ≤ 7.45	pH > 7.45
*N*	64	281	55
Age (years)	65 ± 10	63 ± 13	62 ± 12
Sex male, *n* (%)	37 (58)	158 (56)	32 (58)
ASA classification, *n* (%):			
ASA 1	2 (3)	6 (2)	0 (0)
ASA 2	17 (27)	102 (36)	21 (38)
ASA 3	40 (63)	151 (54)	26 (47)
ASA 4	5 (8)	22 (8)	8 (15)
Indication for HDU admission, *n* (%):			
Type of surgery	42 (66)	194 (69)	39 (71)
Patient comorbidities	22 (34)	87 (31)	16 (29)
Surgical blood loss (ml)	1141 ± 1350	772 ± 958	694 ± 1023
Infusion fluids^a^:			
Balanced crystalloid (Sterofundin®, ml/kg)	37 ± 23	33 ± 20	31 ± 18
Unbalanced crystalloid (0,9% NaCl, ml/kg)	12 ± 10	9 ± 7	8 ± 6
Balanced colloid (Volulyte®, ml/kg)	6 ± 8	4 ± 6	4 ± 6
Unbalanced colloid (Voluven®, ml/kg)	2 ± 4	2 ± 4	1 ± 3
Acid-base parameters:			
pH	7.31 ± 0.03	7.40 ± 0.03	7.48 ± 0.02
pCO_2_ (kPa)	6.0 ± 1.0	5.6 ± 0.6	5.1 ± 1.0
HCO_3_^−^ (mmol/L)	22.6 ± 3.6	26 ± 3.0	28.2 ± 5.4
Sodium (mmol/L)	140 ± 4	139 ± 3	139 ± 5
Chloride (mmol/L)	110 ± 6	107 ± 4	105 ± 6
Lactate (mmol/L)	1.8 ± 1.9	1.1 ± 0.7	1.2 ± 0.6
Albumin (g/L)	28 ± 6	30 ± 6	30 ± 6
BE (mmol/L)	− 4 ± 3	1 ± 3	4 ± 4
AG corrected (mmol/L)	14 ± 4	12 ± 2	11 ± 3
SID apparent (mmol/L)	36 ± 4	38 ± 3	39 ± 7
SID effective (mmol/L)	33 ± 4	37 ± 4	39 ± 6
SIG (mmol/L)	3 ± 3	1 ± 2	0 ± 2
Length of stay (days)	15 ± 11	13 ± 10	13 ± 10
AKI, *n* (%)	10 (16)	12 (4)	0 (0)
Pulmonary embolism, *n* (%)	3 (5)	5 (2)	2 (4)
Myocardial infarction, *n* (%)	1 (2)	1 (0)	3 (6)
Unplanned ICU admission, *n* (%)	14 (22)	25 (9)	5 (9)
Mortality, *n* (%)	8 (13)	6 (2)	2 (4)

*ASA* American Society of Anesthesiologists physical status classification, *HDU* high dependency unit, *BE* base excess, *AG corrected* anion gap corrected for albumin, *SID* strong ion difference, *SIG* strong ion gap, *AKI* acute kidney injury, *ICU* intensive care unit. ^a^Infusion fluids, cumulative fluid therapy from beginning of surgical procedure up to morning after surgery. Data presented as mean ± SD unless otherwise noted

**Table 2:** Acid-base analysis

	pH < 7.35	7.35 ≤ pH ≤ 7.45	pH > 7.45
*N*	64	281	55
Respiratory analysis			
Normocapnia, *n* (%)	42 (66)	238 (85)	42 (76)
Hypercapnia, *n* (%)	18 (28)	20 (7)	2 (4)
Hypocapnia, *n* (%)	4 (6)	23 (8)	11 (20)
Stewart analysis			
SIDa decreased, *n* (%):	37 (58)	101 (36)	17 (31)
Hyperchloremia	25	66	10
Hyperlactatemia	1	5	1
Hyperchloremia + hyperlactatemia	11	11	0
Hyponatremia	0	12	4
Other	0	7	2
SIDa normal, *n* (%)	25 (39)	170 (61)	33 (60)
SIDa increased, *n* (%):	2 (3)	10 (4)	5 (9)
Hypochloremia	1	4	2
Hypernatremia	0	0	0
Other	1	6	3
SIG increased, *n* (%):	23 (36)	60 (21)	6 (11)
Total weak acids, *n* (%):			
Hyperphosphatemia	3 (5)	7 (2)	1 (2)
Hypoalbuminemia:	36 (56)	137 (49)	25 (55)
Traditional analysis			
BE decreased, *n* (%):	33 (52)	16 (6)	1 (2)
AG corrected increased	28	11	0
BE normal, *n* (%):	31 (48)	215 (77)	25 (46)
AG corrected increased	13	81	8
BE increased, *n* (%):	0 (0)	50 (18)a	29 (53)
AG corrected increased	0	15	9

*SIDa* apparent strong ion difference, *SIG* strong ion gap, *BE* base excess, AG corrected, anion gap corrected for albumin

**Fig. 2:**
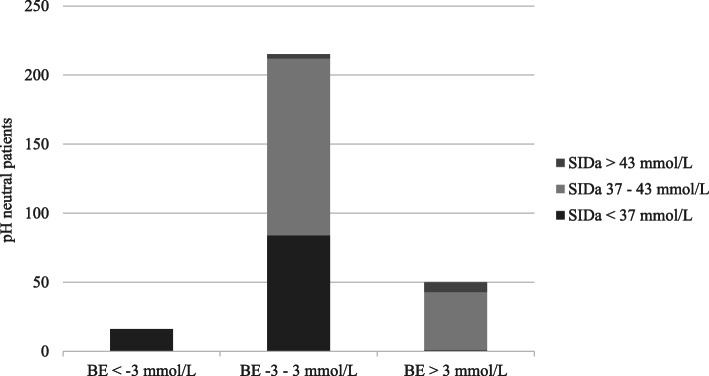
Distribution of decreased (< − 3 mmol/L), normal (− 3–3 mmol/L) and increased base excess (BE) and decreased (< 37 mmol/L), normal (37–43 mmol/L) and increased apparent strong ion difference (SIDa) in pH neutral patients

In pH neutral patients, acidifying changes according to Stewart were frequent; there were 101 cases (36%) with a lowered SIDa of which 84 had normal BE. BE was lowered in 16 cases (6%) of which all had a lowered SIDa. There were 134 pH neutral cases with normal BE as well as AG, of which 58 (43%) had a lowered SIDa. Out of these 58 cases, hyperchloremia contributed to the lowered SIDa in 43 (74%), hyperlactatemia in 9 (17%), and hypoalbuminemia was present in 33 (57%). There were 136 pH neutral cases with normal SIDa and SIG, of which none had a lowered BE while 28 showed an increased corrected AG (21%). Figure 2 shows the distribution of different BE and SIDa categories in pH neutral patients.

In the 46 patients with pH < 7.35 in which hypercapnia did not contribute to the acidemic state, metabolic acidifying disturbances according to Stewart were a lowered SIDa in 34 cases (74%) and increased SIG in 19 (41%) of which 8 with normal SIDa, and when using the traditional approach, a lowered BE in 32 (70%) and increased AG in 35 (76%) of which 8 with normal BE. In the 44 alkalemic patients without hypocapnia, the following metabolic changes contributing to the raised pH were detected: raised SIDa in 5 (11%), lowered total weak acids in 19 (43%). BE was increased in 29 cases (66%).

Several clinical factors were associated with the witnessed acid-base abnormalities. Patients with evidence of kidney dysfunction had a significantly higher SIG and corrected AG then those with serum creatinine < 150 μmol/L (SIG: 1 mmol/L vs. 3 mmol/L, *P* < 0.001. AG corrected: 12 mmol/L vs. 15 mmol/L, *P* < 0.001). Hyperchloremia was associated with increased perioperative use of 0.9% NaCl solution (0.9% NaCl: 10 ml/kg vs. 8 ml/kg, *P* = 0.022). Clinical parameters associated with hypoalbuminemia are shown in Table 3. SIDa and BE were significantly lower in patients with hypoalbuminemia compared to those with normal albumin levels (SIDa: 36 mmol/L vs. 39 mmol/L, *P* < 0.001. BE: 0 mmol/L vs. 1 mmol/L, *P* = 0.009).

**Table 3:** Clinical factors associated with hypoalbuminemia

	Albumin > 30 g/L	Albumin < 30 g/L	
***N***	201	199	
**Surgical blood loss (ml)**	472 ± 653	1167 ± 1234	*P* < 0.001
**Infusion fluids**^**a**^			
Balanced crystalloid (Sterofundin®, ml/kg)	30 ± 19	38 ± 21	*P* < 0.001
Unbalanced crystalloid (0,9% NaCl, ml/kg)	7 ± 5	11 ± 9	*P* < 0.001
Balanced colloid (Volulyte®, ml/kg)	2 ± 4	6 ± 8	*P* < 0.001
Unbalanced colloid (Voluven®, ml/kg)	1 ± 3	3 ± 5	*P* < 0.001

^a^Infusion fluids, cumulative fluid therapy from beginning of surgical procedure up to morning after surgery. Data presented as mean ± SD

Mean LOS was 13 ± 10 days. Increased LOS was associated with changes in SIDa, albumin and corrected AG, but not BE, as shown in Table 4. In patients with neutral pH but with low SIDa as well as those with neutral pH and increased AG, the association with a longer LOS persisted (SIDa: 15 days vs. 12 days, *P* = 0.014. AG: 16 days vs. 12 days, *P* = 0.002). Table 5 shows the diagnostic performance of BE and SIDa for prediction of AKI, unplanned ICU admission, and mortality. Myocardial infarction and pulmonary embolism were too infrequent for any further analysis to be performed.

**Table 4:** Acid-base parameters in association with length of stay in days

pH	> 7.35	< 7.35	
LOS (days)	13 ± 10	15 ± 11	*P* = 0.166
**BE**	**> − 3 mmol/L**	**< − 3 mmol/L**	
LOS (days)	13 ± 10	14 ± 10	*P* = 0.736
**SIDa**	**> 37 mmol/L**	**< 37 mmol/L**	
LOS (days)	12 ± 10	15 ± 10	*P* = 0.015
**AG**	**< 12 mmol/L**	**> 12 mmol/L**	
LOS (days)	11 ± 9	16 ± 12	*P* < 0.001
**SIG**	**< 2 mmol/L**	**> 2 mmol/L**	
LOS (days)	13 ± 11	15 ± 11	*P* = 0.089
**Albumin**	**> 30 g/L**	**< 30 g/L**	
LOS (days)	10 ± 8	16 ± 12	*P* < 0.001

*LOS* length of stay, *BE* base excess, *SIDa* apparent strong ion difference, *AG* anion gap corrected for albumin, *SIG* strong ion gap. Data presented as mean ± SD

**Table 5:** Diagnostic performance of base excess and apparent strong ion difference for prediction of adverse events

	Acute kidney injury	Unplanned ICU admission	Mortality
**BE**, AUC (95% CI)	0.696 (0.597–0.795)	0.647 (0.560–0.733)	0.681 (0.553–0.809
**SIDa**, AUC (95% CI)	0.646 (0.538–0.753)	0.632 (0.541–0.722)	0.686 (0.656–0.807)

*BE* base excess, *SIDa* apparent strong ion difference, *AUC* area under the curve of receiver operating characteristics curve, *ICU* intensive care care unit

## Discussion

This study shows that while the majority of postoperative patients in our population exhibit a neutral pH with pCO_2_ and BE in reference range, underlying metabolic acid-base disturbances are frequent when applying the Stewart approach and the albumin-corrected AG. The two most prominent disturbances according to Stewart are the acidifying effect of a lowered SIDa caused by hyperchloremia and the alkalizing effect of hypoalbuminemia. The concurrent presence of these two metabolic abnormalities can balance each other out and thus result in an apparently normal acid base state as defined by the traditional criteria; however, classifying these cases as normal seems inappropriate given the underlying abnormalities. This result matches the findings of a study on metabolic disturbances in 137 apparent acid-base neutral ICU patients (Moviat et al. 2013).

In our study, the incidence and severity of hypoalbuminemia is constant across the spectrum of pH, with an albumin level of 30 g/L amounting to an alkalizing effect on the BE of approximately + 3 mmol/L (Story 2016). Acidifying metabolic factors oppose the alkalizing effect of hypoalbuminemia with the incidence and magnitude of these effects (low SIDa, increased SIG and corrected AG) increasing as pH shifts from alkalemia to neutral to acidemia. As witnessed in our study, patients presenting with normal pH and pCO_2_ can thus have either no or opposing metabolic changes, either way resulting in a normal BE.

Previous studies addressing postoperative acid-base balance and outcome have focused primarily on metabolic acidosis while defining this as a decrease in BE, with decreased BE due to hyperlactatemia associated with increased mortality (Silva Jr. et al. 2016; Rutherford et al. 1992). In our study, we used LOS as outcome measure as well as the occurrence of major morbid events (e.g., AKI, unplanned ICU admission, mortality). BE and SIDa performed equally in predicting these adverse outcomes. However, changes in BE were not associated with an increase in LOS whereas a decrease in SIDa and increase in corrected AG were, even within pH neutral patients. Aside from providing insight in the mechanisms involved in acid-base balance, this provides further argument as to evaluate the independent metabolic variables defined by Stewart (i.e., SIDa, unmeasured strong anions and albumin) instead of only the dependent variables pH and BE when confronted with ABG results. Whether one uses the AG or SIG as to evaluate the presence of unmeasured strong anions seems a matter of personal preference as these parameters correlate very well (Moviat et al. 2003). Disturbances in SIDa and SIG or AG are most likely a marker of underlying illness rather than the cause of increased LOS by itself and should prompt the clinician to further investigate.

The association of hypoalbuminemia with increased LOS is not surprising as hypoalbuminemia is commonly recognized as a marker for severity of illness and is associated with an unfavorable prognosis (Gibbs et al. 1999; Herrmann et al. 1992). Among its causes are impaired synthesis due to malnutrition, chronic inflammation or critical illness and alterations in distribution due to major surgery or (hemo)dilution (Gatta et al. 2012), with the latter being a likely cause in our population as evidenced by a significantly greater amount of surgical blood loss and subsequently intravenous fluids infused in those with hypoalbuminemia compared to those without.

The observed hyperchloremia causing the low SIDa is most likely iatrogenic in nature due to the administration of 0.9% NaCl solution in the intra- and postoperative period as hyperchloremic patients received a greater amount of 0.9% NaCl, although an adaptive renal response to the alkalizing effect of hypoalbuminemia could be an additional explanation. Whether low SIDa is only a marker of associated hypoalbuminemia, thereby explaining the increase in LOS, or whether there is an additive effect on outcome is unclear. Excess chloride has been associated with increased renal vasoconstriction, decreased urine output, and increased mortality and length of stay after noncardiac surgery (Reid et al. 2003; Wilkes et al. 2001; McCluskey et al. 2013). However, in meta-analysis, when comparing the use of balanced versus non-balanced intravenous solutions in the perioperative period, although the incidence of hyperchloremia was significantly higher in those receiving unbalanced solutions, no difference was found in mortality or major morbidity (Bampoe et al. 2017). Although the consequences of hyperchloremia may be subtle, it seems reasonable to avoid any iatrogenic hemostatic imbalance by reducing the use of 0.9% NaCl solution.

Some limitations of this study should be noted. The incidence of hypoalbuminemia is high in our population due to selection bias and can not be extrapolated to the complete postsurgical HDU population, since only those cases in which the attending physician chose to measure albumin were included. Furthermore, while the crucial determinants of SIDa (sodium, chloride, lactate) were measured by the same blood gas analyzer in one sample, analysis of albumin and other electrolytes were performed on a separate sample in our clinical laboratory. Due to the retrospective nature of the study, the exact time difference between samples is unknown, but should be less than 10 min in the majority of cases, as blood sampling is part of the nursing routines in the morning. This and the fact that magnesium, calcium, and phosphate were not measured and therefore imputed as normal in a minority of patients could have resulted in inaccuracies in the Stewart calculations. Due to its reliance on multiple measurements, the Stewart approach has been said to be vulnerable to inaccuracies, which could provide an alternative explanation for the low SIDa found in our study, especially in those cases in which no overt hyperchloremia or hyperlactatemia was present (Nguyen et al. 2009). However, from clinical experience, we know that calcium and magnesium in most patients show a tendency to be low-normal or below normal. This implies that the results of our calculations of SIDa in cases with missing data might be less pronounced than they would be in reality. Furthermore, magnesium and calcium are considered of minor importance in SIDa variation and are often left out or replaced with normal values in simplified Stewart-based calculations (Story 2016; Agrafiotis et al. 2018).

In order to be able to correct metabolic disturbances, one first needs to identify its existence. In routine clinical care, ABG results are primarily analyzed by observation of pH, BE/HCO_3_^−^, and pCO_2_, while the Stewart approach is often regarded as overly complex and offering no advantage over this method (Emmett 2016). However, our study shows that augmenting the traditional approach with the SIDa leads to increased identification of clinically relevant metabolic disturbances, also in patients in which all traditional parameters are within reference range. These disturbances could otherwise easily be missed. Furthermore, a simplified version of SIDa can be easily calculated at the bedside using only the information provided by the blood gas analyzer.

In conclusion, when applying the Stewart approach and albumin-corrected AG, metabolic disturbances hidden beneath the surface in the patient with seemingly neutral acid-base balance based on pH and BE are frequently detected, can be interpreted within the clinical context, and are associated with increased LOS.

## Data Availability

The dataset used and analyzed during the current study are available from the corresponding author on reasonable request.

## Abbreviations

ASA: American Society of Anesthesiologists
AKI: Acute kidney injury
AG: Anion gap
SIDa: Apparent strong ion difference
AUC: Area under the curve
ABG: Arterial blood gas
BE: Base excess
SIDe: Effective strong ion difference
HDU: High-dependency unit
ICU: Intensive care unit
LOS: Length of stay
ROC: Receiver operating characteristic
SID: Strong ion difference
SIG: Strong ion gap
